# Using Social Vulnerability Indices to Predict Priority Areas for Prevention of Sudden Unexpected Infant Death in Cook County, IL: Cross-Sectional Study

**DOI:** 10.2196/48825

**Published:** 2024-08-20

**Authors:** Daniel P Riggins, Huiyuan Zhang, William E Trick

**Affiliations:** 1 Center for Health Equity & Innovation, Cook County Health, 1950 W Polk St, Suite 5807, Chicago, IL, 60612, United States, 1 773-280-5588; 2 Program in Public Health, Feinberg School of Medicine, Northwestern University, Chicago, IL, United States

**Keywords:** infant, socioeconomic disparities in health, sudden unexpected infant death, SUID, sudden infant death, SID, geographic information systems, structural racism, predict, social vulnerability, racial disparity, socioeconomic, disparity, child, infancy, pediatric, sudden infant death syndrome, SIDS

## Abstract

**Background:**

The incidence of sudden unexpected infant death (SUID) in the United States has persisted at roughly the same level since the mid-2000s, despite intensive prevention efforts around safe sleep. Disparities in outcomes across racial and socioeconomic lines also persist. These disparities are reflected in the spatial distribution of cases across neighborhoods. Strategies for prevention should be targeted precisely in space and time to further reduce SUID and correct disparities.

**Objective:**

We sought to aid neighborhood-level prevention efforts by characterizing communities where SUID occurred in Cook County, IL, from 2015 to 2019 and predicting where it would occur in 2021‐2025 using a semiautomated, reproducible workflow based on open-source software and data.

**Methods:**

This cross-sectional retrospective study queried geocoded medical examiner data from 2015‐2019 to identify SUID cases in Cook County, IL, and aggregated them to “communities” as the unit of analysis. We compared demographic factors in communities affected by SUID versus those unaffected using Wilcoxon rank sum statistical testing. We used social vulnerability indicators from 2014 to train a negative binomial prediction model for SUID case counts in each given community for 2015‐2019. We applied indicators from 2020 to the trained model to make predictions for 2021‐2025.

**Results:**

Validation of our query of medical examiner data produced 325 finalized cases with a sensitivity of 95% (95% CI 93%‐97%) and a specificity of 98% (95% CI 94%‐100%). Case counts at the community level ranged from a minimum of 0 to a maximum of 17. A map of SUID case counts showed clusters of communities in the south and west regions of the county. All communities with the highest case counts were located within Chicago city limits. Communities affected by SUID exhibited lower median proportions of non-Hispanic White residents at 17% versus 60% (*P*<.001) and higher median proportions of non-Hispanic Black residents at 32% versus 3% (*P*<.001). Our predictive model showed moderate accuracy when assessed on the training data (Nagelkerke *R*^2^=70.2% and RMSE=17.49). It predicted Austin (17 cases), Englewood (14 cases), Auburn Gresham (12 cases), Chicago Lawn (12 cases), and South Shore (11 cases) would have the largest case counts between 2021 and 2025.

**Conclusions:**

Sharp racial and socioeconomic disparities in SUID incidence persisted within Cook County from 2015 to 2019. Our predictive model and maps identify precise regions within the county for local health departments to target for intervention. Other jurisdictions can adapt our coding workflows and data sources to predict which of their own communities will be most affected by SUID.

## Introduction

### Background

Sudden unexpected infant death (SUID) exerts a severe burden on affected families and society at large. The National Institute of Child Health and Human Development (NICHD) defines SUID as “the death of an infant younger than 1 year of age that occurs suddenly and unexpectedly.” Sudden infant death syndrome (SIDS) is a specific type of SUID where a cause cannot be identified after a full medical investigation has been completed [1]. Other types of SUID where a cause *can* be identified include but are not limited to “suffocation, mechanical asphyxia, entrapment, infection, ingestions, metabolic diseases, or trauma” [2]. SIDS as a single subtype was ranked as the third leading cause of infant death in the United States in 2020 [3]. In terms of years of life lost (YLL), SIDS accounted for a higher burden of disease than congenital heart anomalies for infants younger than 1 year of age in the United States in 2019 (3600 YLL per 100,000 infants vs 3134) [4].

SUID trends in the US have changed dynamically over time. The respective rates of SUID and SIDS fell from 155 and 130 (cases per 100,000 live births) in 1990 to 90 and 52 in 2010 [5]. Epidemiologists attribute these drops in incidence to a series of programs started in the 1990s to target safe sleep practices. The programs included the release of specific guidelines by the American Academy of Pediatrics (AAP) and an educational campaign called “Safe to Sleep” [5]. The Safe to Sleep program continues to this day and centers on the idea of ABCs: a baby should sleep *Alone* on their *Back* in a separate *Crib* [6]. This is due to evidence that babies are at increased risk of (A) rebreathing their expired gases when lying in a prone or side-lying position and (B) falling victim to accidental suffocation when sharing a sleep surface with caregivers and bulky bed materials [2].

Despite the success of Safe to Sleep and associated programming, trends from the last decade suggest that novel approaches are needed to make further progress in the prevention of SUID. In 2020, the respective rates for SUID and SIDS remained relatively stagnant at 93 and 38 [5]. Such trends hold true in the local context of Illinois, the home state in which Cook County is located. The rate of SUID remained roughly even in Illinois at ~100 (cases per 100,000 live births) between 2000 and 2018. Furthermore, racial disparities were stark. In the Black infant population, rates were actually observed to increase from a nadir of ~200 in 2009 to ~300 in 2018 [7]. Reducing SIDS in a just, effective manner will require addressing these disparities.

There is active discussion in the scientific literature on how best to adjust prevention strategies. Some researchers argue there remains untapped potential in addressing external factors that are named by AAP guidelines but get less attention than the ABCs of safe sleep. For example, use of a pacifier, breastfeeding, and smoking and alcohol avoidance are all recommended protective practices against SUID that get relatively less attention in educational materials [8]. Pretorius and Rew [9] argue that targeting such factors would be particularly effective if implemented outside of the hospital in the clinic or community, where caregivers have greater time and mental capacity to internalize risk reduction strategies.

An example of such an approach in Chicago, IL, was demonstrated by Rasinski et al [10], when they targeted educational campaigns to Black communities estimated to be most at risk for SIDS. They found mixed results from their quasi-experimental design, where some risk behaviors like usage of an improper sleep surface were decreased but other behaviors like placement of extraneous materials in the sleep environment actually increased.

In response to such mixed results, Matoba and Collins [11] argue that to improve high infant mortality rates—and their racially disparate trends—public health practitioners must broaden their focus beyond individual behaviors to neighborhood factors like poverty, air quality, and crime. Indeed, within Chicago, Guest et al [12] demonstrated that geographic variation in infant mortality was significantly associated at the neighborhood level with racial segregation and unemployment.

To counteract these negative neighborhood influences and reduce the community incidence of SUID in a just manner, public health practitioners must precisely target their interventions in space and time. Without spatiotemporal precision, practitioners risk exacerbating inequity by bolstering communities already well resourced while neglecting the communities with the highest need [13].

### Objectives

In this study of Cook County, IL, we sought to enable a neighborhood-focused prevention approach by creating a semiautomated method to precisely describe where SUID occurred in the recent past (2015‐2019) and to predict where SUID would occur in the near future (2021‐2025) while pointing to social vulnerability indicators as explanatory variables. Previous analyses have not provided sufficient geospatial detail to name specific places within counties to target for highest priority [14-17], so we also sought to afford greater detail by using “communities” as our primary unit of analysis.

While these analytic efforts were specific to Cook County, we aimed for our methods to be as adaptable as possible to other jurisdictions by using open-source software and data whenever possible [18,19]. We intended for these tools to enable any interested jurisdiction to get assistance to communities most afflicted by SUID in a timely, targeted manner.

## Methods

### Study Design

We conducted a cross-sectional retrospective study on case counts of SUID in “communities” of Cook County, IL.

We used the concept of communities in order to increase the geographic catchment area for case counts (incidence in smaller census tracts was too low to make robust case count predictions) as well as to lend semantic meaning to the geographic unit of analysis, that is, being able to name each unit rather than using a numeric identifier.

Since census tracts and existing community boundaries do not exactly align, we designed a method for resolving differences. First, for each census tract, we calculated its centroid (the geographic center of an irregular shape). Second, we sent each census tract’s centroid to the OpenCage service [20,21], which uses open data to assign labels to geographic points (eg, the corresponding country, state, city, suburb, neighborhood, etc, for a given point). Third, we used a decision rule to resolve the collection of multiple labels for a given census tract into a single “community.” A community roughly corresponded to one of the 77 formally defined Chicago Community Areas (if within city limits) [22] or one of Suburban Cook County’s independent municipalities [23]. Finally, we performed a spatial join of all census tracts with the same community label (ie, combine all census tracts together by their shared borders into a common external border).

Although aggregating to “communities” lent the benefits listed above, it came at the cost of less precision for corresponding social vulnerability covariates, which were aggregated into community-level values by taking simple sums of their estimates at the census tract level without accounting for variations in their margins of error.

### Primary Outcome

**Figure 1.**
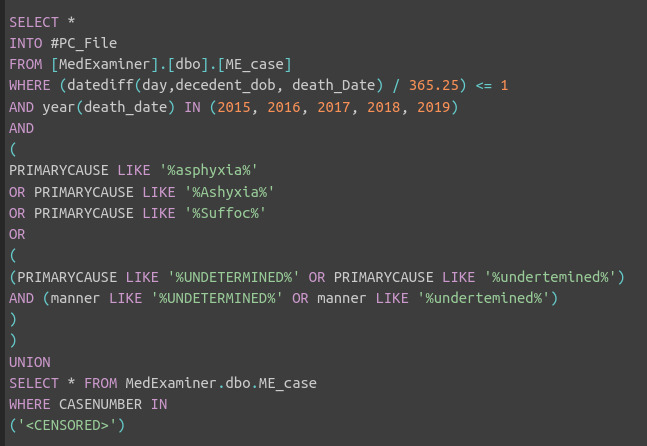
SQL query of the medical examiner’s archive for identifying cases of sudden unexpected infant death (SUID) in communities of Cook County, IL, from 2015 to 2019. The “WHERE CASENUMBER IN” clause is censored here for confidentiality, but it contained a list of additional case numbers recommended for inclusion by team members from the SUID Case Registry for Cook County.

The primary outcome of interest was the SUID case count in each community. We translated the NICHD’s definition of SUID [1] into a structured query language (SQL) query of the Cook County Medical Examiner Office’s Archive (Figure 1). Each case identified by the query was validated by team members from the SUID Case Registry for Cook County, who also shared cases that were missed by the query process. Each validated case was geocoded confidentially with latitude and longitude by using ArcGIS Pro geocoding tools behind Cook County’s firewall. Geocoded cases occurring in each community were aggregated into case counts.

### Temporal Setting

The year of origin for each data variable was context-specific. SUID case counts were observed from 2015 to 2019. A descriptive comparison of census tracts with and without SUID present was performed on variables from contemporaneous years. We retrospectively trained a model predicting SUID case counts from 2015 to 2019 using predictor covariates from 2014. Using the trained model, we made prospective predictions for SUID case counts from 2021 to 2025 with covariates from 2020.

### Coding Pipeline

We performed all steps in the data pipeline downstream from the SQL query in the R computing environment [24] using the “tidyverse” suite of packages [25] (The R Foundation). We used additional data cleaning convenience functions from the “janitor” and “RSocrata” packages [26,27]. The data pipeline was orchestrated using the “targets” package [28]. We generated data tables for publication using the “gt” package and its companion {gtsummary} [29,30]. We performed geospatial manipulation and mapping with the “sf,” “leaflet,” “tmap,” “opencage,” and “tigris” packages [31-34] along with their underlying computational infrastructure [35-41].

### Statistical Analysis

#### Descriptive Comparison

We compared communities with and without observed cases of SUID using median values (and IQRs) of demographic variables. Median values were reported because most of the variables did not approximate normal distributions. Comparisons between communities with and without observed SUID were statistically conducted with the Wilcoxon rank sum test.

#### Predictive Modeling

We sourced all predictive covariates from US Census’ “American Community Survey” (ACS) and from the Agency for Toxic Substances and Disease Registry’s associated “Social Vulnerability Index” (SVI), which has 4 thematic domains: Socioeconomic (eg, estimate of people living below the poverty line), Household Composition and Disability (eg, estimate of single-parent households with children under 18), Minority Status and Language (eg, estimate of all non-White, non-Hispanic people), and Housing Type and Transportation (eg, estimate of households with more occupants than rooms) [42,43].

We modeled SUID case counts in each census tract using maximum likelihood estimation via the “MASS” R package [44]. We used the negative binomial family of generalized linear models instead of the Poisson family because overdispersion was detected [45]. A random forest model was considered as an alternative to negative binomial, but stakeholders expressed preference for a model where results could be explained by explicit predictor variables and their covariates.

The selection of model forms and predictors is described in detail in Multimedia Appendix 1. Briefly, we started with the total population as an offset variable for use in the model. Next, to consider other predictor candidates, we calculated the Pearson correlation coefficients between the SUID case count and each underlying variable in the SVI [43]. One to two promising variables from each SVI thematic domain were assessed in the model using an additive step-wise strategy. The performance parameters assessed were Akaike information criteria and Bayesian information criteria, root mean squared error, and Nagelkerke *R*^2^ using the “EasyStats” suite of R packages [46]. All predictor variables were log-transformed in the final model in order to reduce the influence of extreme values.

### Ethical Considerations

To validate our query of the Cook County medical examiner’s database, infant mortality in the medical examiner data was shared with the county’s infant mortality review panel, who already had access to these data. As a population health study using publicly available data sources on decedents, this study was exempt from institutional review board review and informed consent processes. The analytic dataset was deidentified, and for publication, data were aggregated to protect affected families.

### Data Availability

An export of the analytic dataset is available in Multimedia Appendix 2 or in an open web-based SQL database on DoltHub [19].

## Results

### Case Identification

Our SQL query identified 333 prospective cases of SUID from the medical examiner archives. Reviewers from the SUID Case Registry of Cook County recommended adding 16 cases and removing 7 cases. Using all cases of death in children under 1 year of age as the denominator, our electronic query achieved a sensitivity of 326/342 (95%; 95% CI 93%‐97%) and a specificity of 102/109 (98%; 95% CI 94% to 100%). One case could not be used in subsequent spatial analyses because there was no associated address, which led to a final case count of 325.

### Geospatial Aggregation

We spatially aggregated cases into counts within 199 communities; 49% of communities (97/199) observed at least one case of SUID from 2015 to 2019. Table 1 shows the full distribution of case counts.

**Table 1.** Cross-sectional distribution of sudden unexpected infant death (SUID) case counts in communities of Cook County, IL, from 2015 to 2019.

SUID case count	Frequency, n (%)
0	102 (51.3)
1	36 (18.1)
2	24 (12.1)
3	8 (4.0)
4	7 (3.5)
5	3 (1.5)
6	4 (2.0)
7	5 (2.5)
8	1 (0.5)
9	2 (1.0)
10	3 (1.5)
11	1 (0.5)
12	0 (0)
13	0 (0)
14	1 (0.5)
15	0 (0)
16	0 (0)
17	2 (1.0)

### Mapping SUID Case Counts

**Figure 2.**
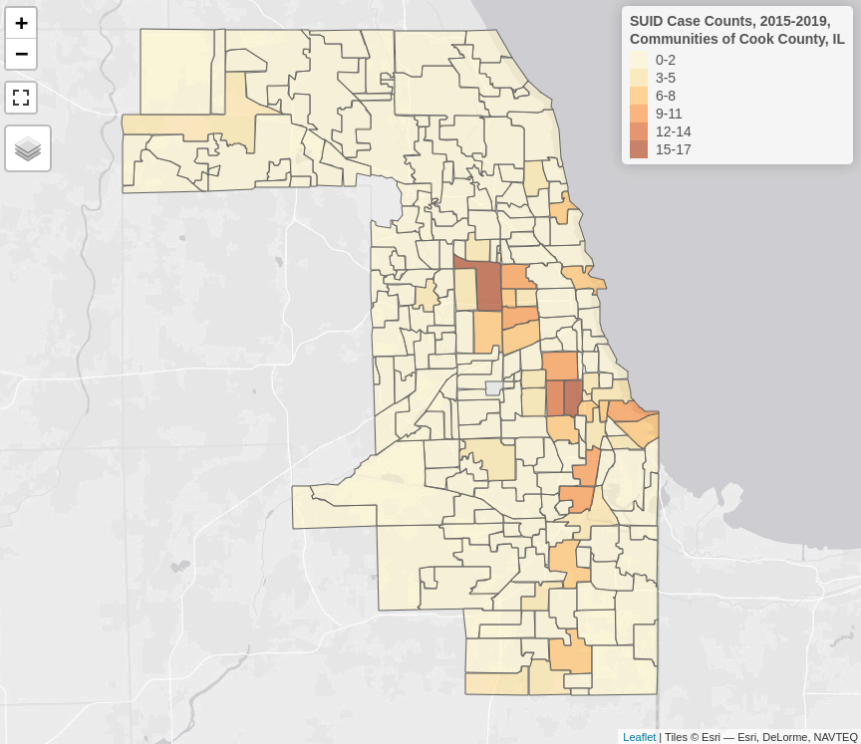
Snapshot of an interactive map of cross-sectional case counts of sudden unexpected infant death (SUID) in communities of Cook County, IL, from 2015 to 2019.

We generated an interactive map of SUID case counts (Figure 2), which can be viewed online [47]. The map showed subjective clusters of cases on the West and South Sides of Chicago as well as the South Suburbs of Cook County. The top 10 communities with the largest case counts were all located within Chicago city limits: Austin (17 cases), Englewood (17 cases), West Englewood (14 cases), West Pullman (11 cases), Humboldt Park (10 cases), New City (10 cases), Roseland (10 cases), North Lawndale (9 cases), South Shore (9 cases), and the Near North Side (8 cases). The community outside of Chicago city limits with the largest case count was Chicago Heights (7 cases).

### Comparing Communities Affected Versus Unaffected by SUID

**Figure 3.**
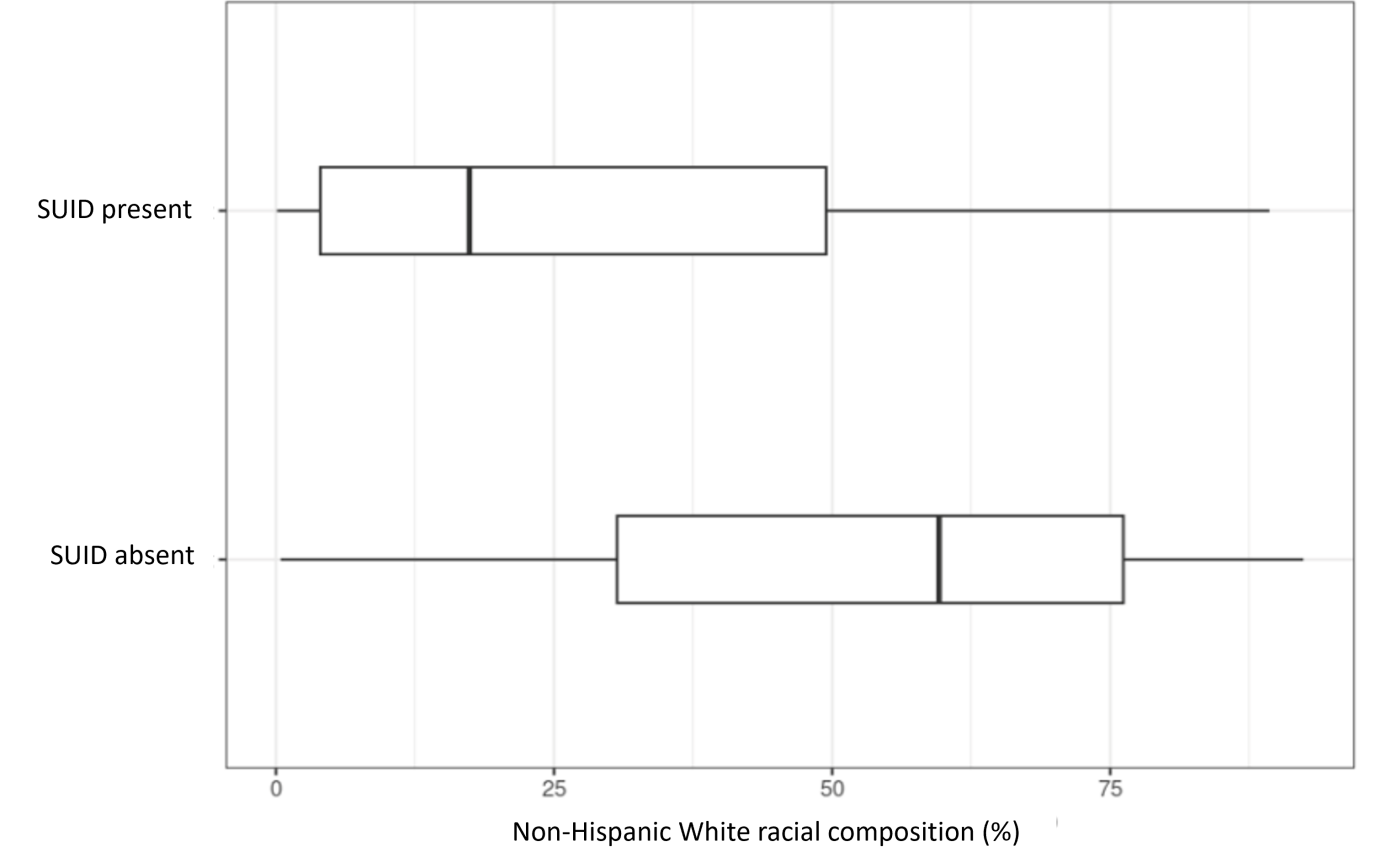
A cross-sectional comparison of White racial composition in communities of Cook County, IL, affected versus unaffected by sudden unexpected infant death (SUID) from 2015 to 2019.

**Figure 4.**
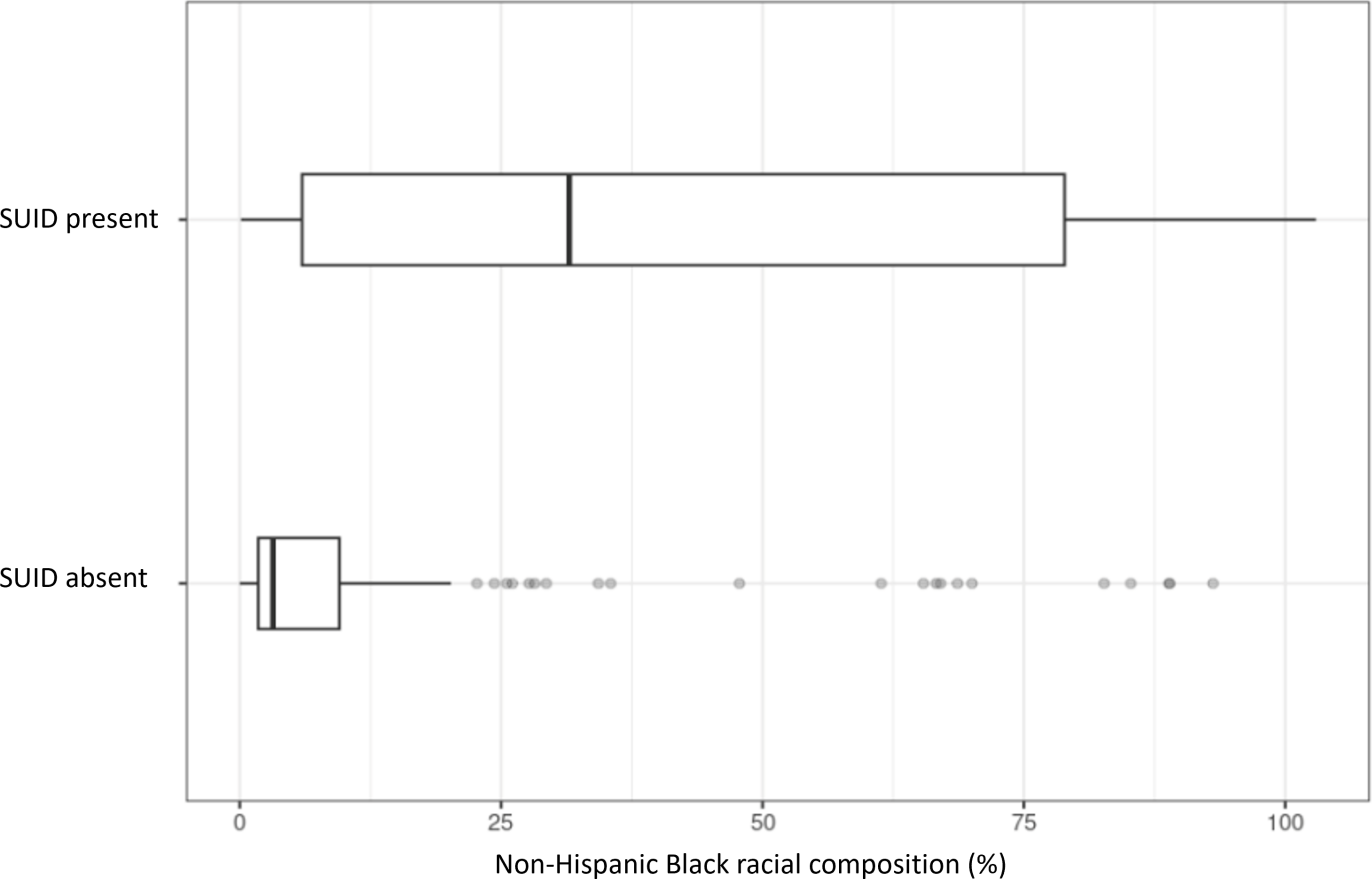
A cross-sectional comparison of Black racial composition in communities of Cook County, IL, affected versus unaffected by sudden unexpected infant death (SUID) from 2015 to 2019.

Table 2 compares the demographics of communities observed with at least one case of SUID versus those without. There were statistically significant differences in age and sex composition, although the absolute differences were slight. In terms of race, communities with SUID present exhibited a lower composition of Asian residents at 2% versus 5% (*P*<.001) and non-Hispanic White residents at 17% versus 60% (*P*<.001; Figure 3). Conversely, communities with SUID present exhibited a higher composition of non-Hispanic Black residents at 32% versus 3% (*P*<.001; Figure 4). There was no statistically significant difference in Hispanic composition. Variables algorithmically selected for use in the predictive model all showed statistically significant differences as well.

**Table 2.** A cross-sectional comparison of demographics in communities of Cook County, IL, affected versus unaffected by sudden unexpected infant death (SUID) from 2015 to 2019.

Variable		SUID present (n=97), median (IQR)	No SUID (n=102), median (IQR)	*P* value^a^
**Age**				
	Median age (years)	37 (34-40)	40 (36-43)	<.001
**Sex**				
	Sex ratio (males per 100 females)	91 (84-97)	96 (91-101)	.001
**Race or ethnicity**				
	American Indian and Alaska Native, any (%)	1 (0-1)	1 (0-1)	.05
	Asian, any (%)	2 (1-6)	5 (2-11)	<.001
	Non-Hispanic Black, alone (%)	32 (6-79)	3 (2-10)	<.001
	Non-Hispanic White, alone (%)	17 (4-49)	60 (31-76)	<.001
	Hispanic, any (%)	14 (4-28)	14 (7-33)	.20
**Modeling variables**				
	Total population	23,810 (13,811-47,181)	14,426 (8446-26,577)	<.001
	Total people living below poverty	4,354 (1873-8421)	1252 (578-2692)	<.001
	Total crowded households	284 (116-639)	116 (38-330)	<.001

a Wilcoxon rank sum test.

### Modeling SUID Case Counts

**Figure 5.**
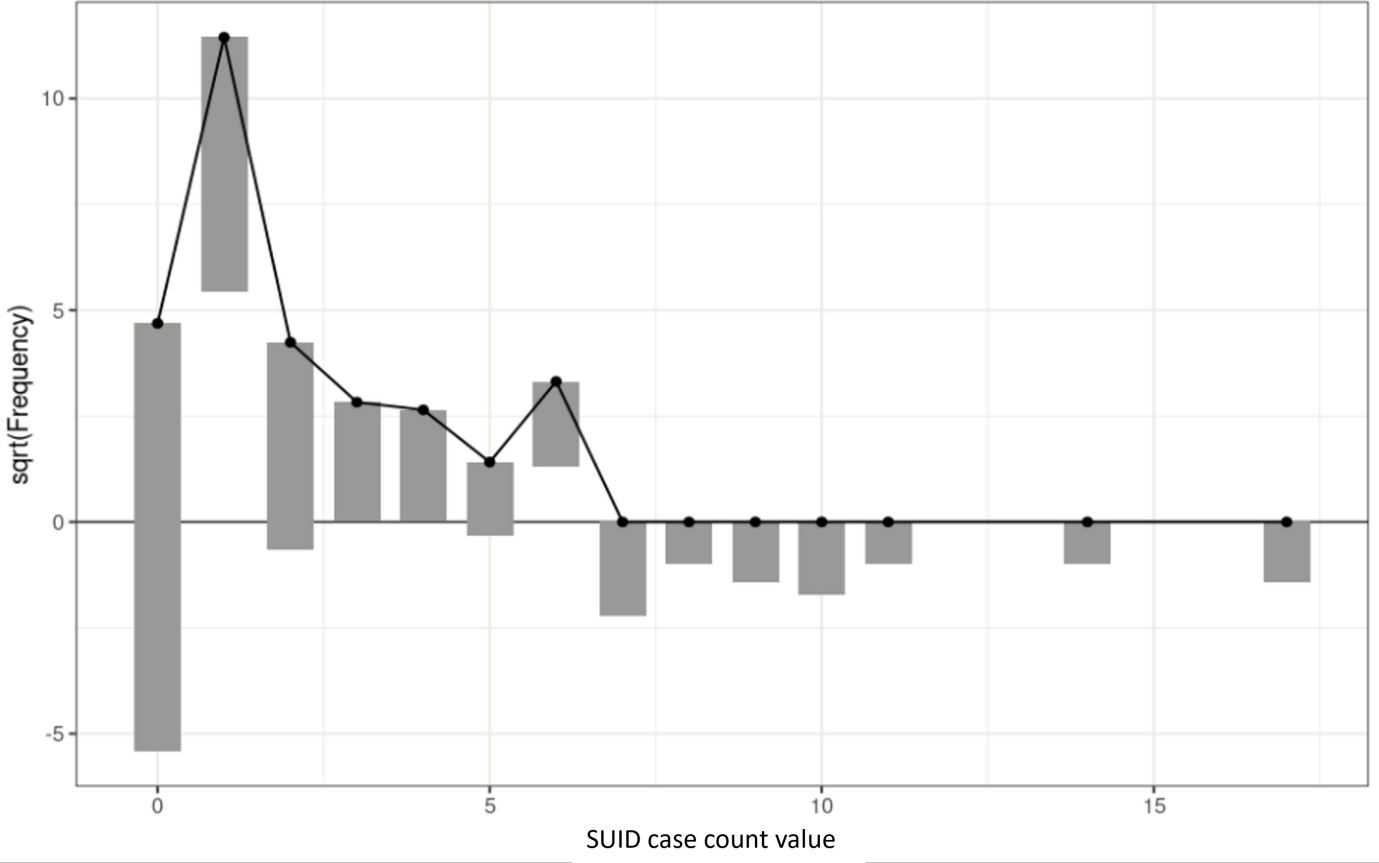
Rootogram depicting goodness of fit for a negative binomial regression model predicting case counts of sudden unexpected infant death (SUID) in communities of Cook County, IL. Training data used outcome case counts from 2015 to 2019 and predictor variables from 2014. Observed case counts are the bars “hanging” from predicted case counts “pinned” at the dots. For aggregate underpredictions, the bar hangs below zero; for aggregate overpredictions, the bar hangs above zero.

We fit a negative binomial regression model to predict SUID case counts for each community of Cook County based on total population, total people living below the poverty line, and total households with more occupants than rooms (“crowded” households; Table 3). The model was retrospectively trained on SUID case counts for 2015‐2019 with predictor variables from the 2014 SVI and ACS. Figure 5 depicts goodness of fit for the model on the training data, showing that it captured mid-range counts well, but underpredicted the number of communities with zero cases, overpredicted those with one case, and underpredicted those with counts over 6. Evaluation of the model’s predictive performance showed a Nagelkerke *R*^2^ value of 70.2% and a root mean squared error of 17.49. See Multimedia Appendix 1 for further details on evaluation of performance.

The median difference between predicted and observed case counts in the training data was 0.24 (IQR −0.57 to 0.60). All 5 communities with the highest observed case counts (Austin, Englewood, Humboldt Park, West Englewood, and West Pullman) were also among the communities with the most significant individual underpredictions (ranging from 4.05 to 7.61 less than that observed). On the other end of the spectrum, the 5 communities with the most significant individual overpredictions were Chicago Lawn, Douglas, Grand Boulevard, Kenwood, and Rogers Park (ranging from 2.94 to 4.77 more than that observed; data available in Multimedia Appendix 2).

**Table 3.** Exponentiated parameters for a negative binomial regression model predicting case counts of sudden unexpected infant death in communities of Cook County, IL.^a^

Parameter	RR^b^	SE	95% CI	z-score	*P* value
Intercept	0.00134	0.00165	1.12e−04 to 0.01	−4.30	<.001
log(Total crowded households)	0.68792	0.07917	0.55 to 0.87	−2.59	.001
log(Total people living below poverty)	6.06547	1.01710	4.42 to 8.44	8.58	<.001
log(Total population)	0.55181	0.09339	0.40 to 0.77	−2.80	<.001

a Training data used outcome case counts from 2015 to 2019 and predictor variables from 2014.

b RR: risk ratio. Interpreted as the magnitude by which you would multiply the risk with a unit increase of 1 for each covariate.

### Prospective Model Predictions

**Figure 6.**
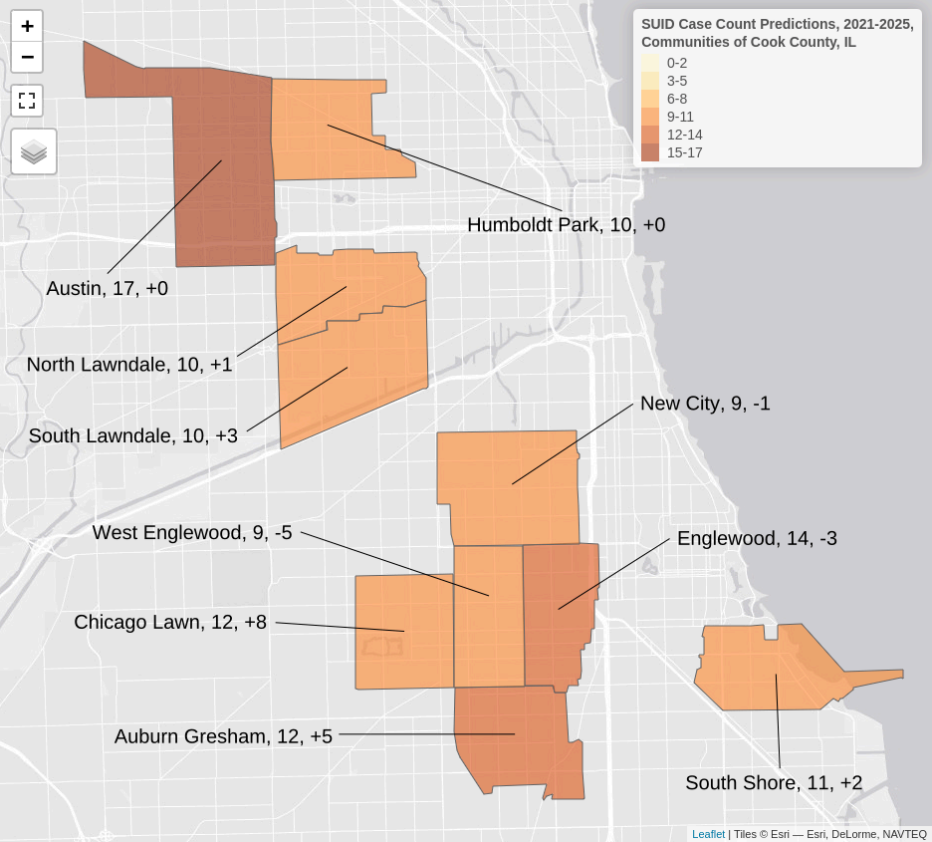
A map of top 10 predicted case counts for a negative binomial regression model predicting case counts of sudden unexpected infant death (SUID) in communities of Cook County, IL, from 2021 to 2025. Labels: community name, predicted case count, predicted change from 2015‐2019.

We applied predictor variables from the 2020 SVI and ACS to our trained model to predict case counts for 2021‐2025. Most of the top 10 communities predicted to have the highest case counts for 2021‐2025 were the same as those observed in the top 10 for 2015‐2019 except for Chicago Lawn, Auburn Gresham, and South Lawndale with predicted changes of +8, +5, and +3, respectively (Table 4). Figure 6 depicts these communities in spatial relation to each other.

**Table 4.** Top 10 predicted case counts for a negative binomial regression model predicting case counts of sudden unexpected infant death in communities of Cook County, IL.^a^

	2021‐2025		2015‐2019		
Community	Predicted count	Predicted change^b^	Predicted count	Observed count	Residual^c^
Austin	17	+0	11	17	−6
Englewood	14	−3	9	17	−8
Auburn Gresham^d^	12	+5	7	7	+0
Chicago Lawn^d^	12	+8	7	4	+3
South Shore	11	+2	8	9	−1
Humboldt Park	10	+0	6	10	−4
North Lawndale	10	+1	8	9	−1
South Lawndale^d^	10	+3	7	7	+0
New City	9	−1	5	10	−5
West Englewood	9	−5	7	14	−7

a Training data used outcome case counts from 2015 to 2019 and predictor variables from 2014. Forward predictions were made for the period from 2021 to 2025 using predictor variables from 2020.

b (Prediction from 2021 to 2025) − (Observation from 2015 to 2019).

c (Prediction from 2015 to 2019) − (Observation from 2015 to 2019).

d New to the top 10 (not in the top 10 for observed case counts from 2015 to 2019).

## Discussion

### Principal Results

Our process for querying data from the Cook County Medical Examiner was able to identify cases of SUID from 2015 to 2019 with a high degree of fidelity to those identified by the county’s official registry. We aggregated these cases to the “community” level and found that those containing a case of SUID during the study period also had higher proportions of Black residents and lower proportions of Asian and White residents. We built a negative binomial regression model to predict SUID case counts and achieved moderate accuracy, especially within the mid-range of case counts. As an aide for deciding where to target preventive services, we mapped the communities most affected by SUID. Our results suggest that intervention programs should focus efforts on the north and west sides of Chicago and in the south suburbs of Cook County. The top 10 communities predicted to have the highest case counts were also in these general areas. Chicago Lawn was predicted to emerge with the largest predicted change of +8 cases.

### Implications

We suggest our analytic outputs should be used in different ways based on their temporal context. Within the model training period of 2014‐2019, residuals between predictions and observations represent promising routes of inquiry for expanding understanding of community-level ecological factors correlating with SUID incidence. For example, public health practitioners might consider intensive qualitative inquiry in the communities most underpredicted (like Austin and Englewood) to identify new risk factors and in communities most overpredicted (like Douglas and Grand Boulevard) to identify new protective factors unaccounted for by the model. After the model training period in 2020‐2025, our prospective predictions represent opportunities to anticipate shifting dynamics in the communities most affected by SUID.

Regardless of the temporal context, all results from this study can be used to guide a targeted, local approach to SUID prevention. Turman and Swigonski [48] proposed a framework for such an approach. Both high-level goals of their framework hinged on identifying specific zip codes with the highest infant mortality rates in Central Indiana. The first goal focused on developing infrastructure in those zip codes to support healthy pregnancies and infants. For example, they increased the capacity of local early childhood education programs, which both provided childcare for mothers seeking employment and served as vehicles for education on safe sleep practices. The second goal focused on training women from those zip codes as grass roots maternal child health leaders. This framework is just as applicable to Cook County as it is to Central Indiana. Many of the key institutional partners like a local Fetal Infant Mortality Review Board are already in place and could use the maps and predictions from this study to identify targeted communities most at risk.

For a variety of reasons, more work must be done before designing interventions to apply within the framework above. First, because our model is designed for prediction rather than causal inference, differences in model covariates should not be interpreted as directly leading to differences in SUID incidence. Hence, we would caution against trying to act on these covariates in isolation. Second, the SVI on its own does not capture many population-level factors that would be directly relevant to modifiable, individual-level factors specific to SUID (eg, safe sleep practice, smoking, alcohol use, illicit drug use, and engagement in prenatal care) [49]. Additional work must be done to acquire data on these factors in our jurisdiction. Finally, one Cochrane synthesis of evidence suggests that a key source of data for health interventions is generated by direct conversation with local stakeholders in communities identified as most affected [50], which has yet to be performed. If local coalitions express preference for a menu of potential options, they might consider deploying targeted digital assessments of in-home sleep environments [51], smoking cessation campaigns, or educational programming in community-based organizations.

### Comparison With Prior Work

Our findings are consistent with the broader body of research on sociodemographics related to infant mortality. Several other studies have also identified a correlation between infant mortality and community-level factors like alcohol or drug use, education, employment, immigration, insurance, involvement in child protective services, poverty, racism, and racial segregation [12,15,16,52-55]. Some studies have posited direct causal relationships between these factors and infant mortality, but the evidence is still equivocal. Both Hearst et al [55] and Johnson et al [54] used propensity score matching as a means of isolating such causal effects, the former for residential segregation on Black infant mortality and the latter for neighborhood poverty on American Indian infant mortality. Both were unable to detect an influence but emphasized that their inability to detect effects may have been due to limitations in the size of sampling pools to achieve adequate counterfactual comparisons. Our study does not attempt to identify causal effects and instead focuses on helping local health departments target precise regions of their jurisdictions for intervention.

Our study advances geospatial research on SUID in 3 major ways. First, we added finer detail to the available Chicago-based maps by using census tracts and “communities” as areal units, with the added benefit that these can be linked to census-derived demographics. Second, we added interactive capability to our maps for greater utility to practitioners wanting to explore the data first-hand. Third, to our knowledge, this is the first study to have made prospective predictions on the spatial incidence of SUID, helping to anticipate changing dynamics in regions of interest. In comparison, Briker et al [14] performed a different but complementary analysis of Cook County data, visualizing SUID incidence in 2015‐2016 by kernel density estimation (KDE). Relative to our method of displaying counts per “community” areal unit, KDE has the advantage of smoothing out random variation observed in the data to make clusters more apparent. With this method, the authors also found strong clusters of SUID on the West and South Sides of Chicago. The comparative disadvantages of KDE are that the visualized kernels do not have one-to-one matches with real-world administrative boundaries, and the units of intensity are less interpretable than counts. Also, because the map in Briker et al was not interactive, it was more difficult to ascertain specific high-risk communities. In another study, Drake et al [15] used KDE to visualize SUID incidence in Harris County, TX, and this approach had the same advantages and disadvantages as described above. Fee and Tarrell [16] used analogous techniques to those used in our study by visualizing incidence in administrative areal units of Douglas County, NE. These authors were also able to correlate incidence with other variables, although their variables were more relevant to individual-level risk (eg prenatal care and tobacco use) rather than the population-level approach used in our study. In an older seminal study, Grimson et al [17] focused on demonstrating statistical methods for identifying geospatial clusters. Their work is relevant to our study because the authors used SUID incidence as their example use-case, but our study was not focused on identifying statistically significant clusters and was more finely detailed on a smaller geographic scale.

### Limitations

Our study has limitations. First, a Durbin-Watson test of our model for autocorrelated residuals rejected the null hypothesis, suggesting that observations were not independent from each other. This was at least partially due to the interconnected nature of census tracts in physical space. Indeed, a Moran I test also rejected the null hypothesis, suggesting the presence of spatial autocorrelation. Second, our model did not account for measurement error in our covariates, which are estimated by the ACS using subsamples of the population. Failure to account for the margins of error and for spatial autocorrelation risked introducing systematic bias into our model predictions [56]. This was likely compounded by aggregating estimates for census tracts into the “community” areal unit. Trade-offs for this approach are described in the *Methods* section.

One means of addressing these first two limitations would be to implement a hierarchical Bayesian model of spatial measurement error [57]. We attempted to do so using the “geostan” R package [58], but preliminary attempts yielded warnings from the software that sampling chains did not converge--significantly raising the risk of inaccurate parameter estimates. An additional limitation, which may have contributed to nonconvergence, was that we did not have access to estimates of the true denominator for the incidence of SUID (the count of live births in each areal unit during the study period). The model implemented in geostan requires an offset denominator variable, so we used population counts of children under 5 as a proxy, but this may have contributed to additional imprecision that hampered the sampling algorithm’s ability to converge.

A final limitation was that not enough time had passed to empirically assess our model’s prospective predictions. Decision makers at local health departments might be more confident in the model’s performance if they could see how it fared at predicting outside of the training dataset. We suggest mitigating this limitation by comparing prospective predictions with retrospective observations, both of which together make a more compelling argument for risk than either piece of information alone.

### Conclusions

Like many public health jurisdictions throughout the United States, Cook County, IL, is mired in persistent rates of SUID tied to racial and socioeconomic disparities. Our semiautomated process for compiling, analyzing, and predicting cases may allow intervention programs to more quickly and effectively address such disparities with efforts targeted at communities most in need of prevention services.

## Supplementary material

10.2196/48825 Multimedia Appendix 1 Model selection and evaluation.

10.2196/48825 Multimedia Appendix 2 Data export.

## Abbreviations

AAP: American Academy of Pediatrics
ABCs: An infant should sleep Alone on their Back in a Crib to promote safe sleep
ACS: American Community Survey
KDE: kernel density estimation
NICHD: National Institute of Child Health and Human Development
SIDS: sudden infant death syndrome
SQL: structured query language
SUID: sudden unexpected infant death
SVI: Social Vulnerability Index
