# Supplementary material for: Using Social Vulnerability Indices to Predict Priority Areas for Prevention of Sudden Unexpected Infant Death in Cook County, IL: Cross-Sectional Study
Source: JMIR Public Health Surveill. 2024 Aug 20;10:e48825. doi: 10.2196/48825 (PMC11350474; doi:10.2196/48825)
Supplement: Multimedia Appendix 1 [file publichealth-v10-e48825-s001.pdf]

Supplement 1: Model Selection and Evaluation

AUTHOR
Daniel P. Hall Riggins

PUBLISHED
April 12, 2023

This supplement lays out how we selected our model for predicting the number of SUID cases per "community" (variable `suid_count_2015_2019`) from variables in the CDC's Social Vulnerability Index (SVI) dataset. Component variables used in the index are divided into four overarching themes: "Socioeconomic", "Household Composition/Disability", "Minority Status/Language", "Housing Type/Transportation".

Start by loading R libraries and the dataset:

```
library(dplyr)
```

```
Attaching package: 'dplyr'

The following objects are masked from 'package:stats':

    filter, lag

The following objects are masked from 'package:base':

    intersect, setdiff, setequal, union
```

```
library(sf)
```

```
Linking to GEOS 3.10.2, GDAL 3.4.1, PROJ 8.2.1; sf_use_s2() is TRUE
```

```
library(performance)

suid <- arrow::read_parquet("data/suid_export.parquet")
```

Choosing Model Type

We explored the general family of models that expect an outcome to be distributed as a count. These distribution types include Poisson, Negative Binomial, and their zero-inflated variants.

Overdispersion

Poisson would have been the simplest model choice, but was unable to account for overdispersion, which was present in our data:

```
glm(
    formula = suid_count_2015_2019 ~ 1,
```

```
    family = poisson(),
    data = suid
) |>
    check_overdispersion()
```

```
# Overdispersion test

       dispersion ratio =    5.165
  Pearson's Chi-Squared = 1022.689
                p-value =  < 0.001

Overdispersion detected.
```

Using a negative binomial model resolved the issue with overdispersion:

```
MASS::glm.nb(
    suid_count_2015_2019 ~ 1,
    data = suid
) |>
    check_overdispersion()
```

```
# Overdispersion test

       dispersion ratio =   1.033
  Pearson's Chi-Squared = 204.532
                p-value =    0.36

No overdispersion detected.
```

Zero-inflation

If a negative binomial model is zero-inflated, one can use a variant of the model to correct for that, but this was not an issue in our context:

```
MASS::glm.nb(
    suid_count_2015_2019 ~ 1,
    data = suid
) |>
    check_zeroinflation()
```

```
# Check for zero-inflation

   Observed zeros: 102
  Predicted zeros: 103
            Ratio: 1.01

Model seems ok, ratio of observed and predicted zeros is within the
  tolerance range.
```

Therefore we settled on a negative binomial model type as our final choice.

Identifying Predictor Candidates

Population Adjustment

Before selecting other predictor variables, was helpful to know if including population estimate as an exposure offset in the model added value.

```
totpop <-
    MASS::glm.nb(
        suid_count_2015_2019 ~ e_totpop_2014,
        data = suid
    )

parameters::parameters(totpop, exponentiate = TRUE, digits = 6) |>
    print_html()
```

Parameter	Coefficient	SE	95% CI	z	p
(Intercept)	0.682601	0.129026	(0.45, 1.03)	-2.020116	0.043
e totpop 2014	1.000027	0.000005	(1.00, 1.00)	5.341993	< .001

In our case, the population offset did have a statistically significant contribution to the model.

Other Predictors

We generated a dataframe containing correlation coefficients for each SVI variable to `suid_count_2015_2019`:

```
suid_correlations <-
    suid |>
    as_tibble() |>
    select(
        suid_count_2015_2019,
        ends_with("_2014"),
        -starts_with("log_e")
    ) |>
    corrr::correlate() |>
    arrange(desc(abs(suid_count_2015_2019)))
```

```
Correlation computed with
• Method: 'pearson'
• Missing treated using: 'pairwise.complete.obs'
```

```
suid_correlations |>
    select(term, suid_count_2015_2019)
```

```
# A tibble: 21 × 2
   term           suid_count_2015_2019
   <chr>                         <dbl>
 1 e_pov_2014                    0.719
 2 e_sngpnt_2014                 0.689
 3 e_unemp_2014                  0.671
 4 e_minrty_2014                 0.631
 5 e_disabl_2014                 0.567
 6 e_uninsur_2014                0.466
 7 e_nohsdp_2014                 0.457
 8 e_age17_2014                  0.434
 9 e_crowd_2014                  0.428
10 e_noveh_2014                  0.415
# ℹ 11 more rows
```

So for example, the correlation between percentage unemployed and SUID count was 0.33.

Sidenote: the SVI dataset prefixes its variables with abbreviations to denote different types. A prefix of "e_" denotes that variable represents a raw estimate as opposed to a percentile ("ep_") or a margin of error ("m_").

Our approach to choosing predictors was to screen performance of models in a step-wise additive fashion. We paid close attention to metrics that penalize for overfitting due to inclusion of too many predictor variables–aka Akaike/Bayesian Information Criteria (AIC; BIC).

To shrink the number of models to screen, we started with the variable from each SVI theme that was most correlated to SUID count. So to start out, we screened the following variables:

Theme 1 - Socioeconomic: `e_pov_2014` - Estimate of people living below the poverty line, 2010-2014 American Community Survey (ACS)

Theme 2 - Household Composition/Disability: `e_sngpnt_2014` - Estimate of single-parent households with children under 18, 2010-2014 ACS

Theme 3 - Minority Status/Language: `e_minrty_2014` - Estimate of minority people (all persons except white, non-Hispanic), 2010-2014 ACS

Theme 4 - Housing Type/Transportation: `e_crowd_2014` - Estimate of households with more occupants than rooms (crowded), 2010-2014 ACS

Here, we fit a model using each of these variables added to total population, then compared performance:

```
totpop_pov <-
    MASS::glm.nb(
        suid_count_2015_2019 ~ e_pov_2014 + e_totpop_2014,
        data = suid
```

```
    )

totpop_sngpnt <-
    MASS::glm.nb(
        suid_count_2015_2019 ~ e_sngpnt_2014 + e_totpop_2014,
        data = suid
    )

totpop_minrty <-
    MASS::glm.nb(
        suid_count_2015_2019 ~ e_minrty_2014 + e_totpop_2014,
        data = suid
    )

totpop_crowd <-
    MASS::glm.nb(
        suid_count_2015_2019 ~ e_crowd_2014 + e_totpop_2014,
        data = suid
    )

compare_performance(
    totpop_pov, totpop_sngpnt, totpop_minrty, totpop_crowd,
    metrics = "common",
    rank = TRUE
) |>
    print_html()
```

Comparison of Model Performance Indices

Name	Model	Nagelkerke's R2	RMSE	AIC weights	BIC weights	Performance-Score
totpop_pov	negbin	0.67	10.97	1.000	1.000	94.61%
totpop_minrty	negbin	0.54	13.96	1.02e-05	1.02e-05	35.39%
totpop_crowd	negbin	0.23	3.00	1.02e-16	1.02e-16	25.00%
totpop_sngpnt	negbin	0.56	39.94	3.54e-05	3.54e-05	18.75%
NA						

The model adding the poverty metric performed best.

In an attempt to find information less co-linear to poverty, we looked for variables in each theme that were most correlated to SUID count that also had “low” correlation to poverty.

```
suid_correlations |>
    filter(abs(e_pov_2014) < 0.25) |>
```

```
    relocate(e_pov_2014, .after = suid_count_2015_2019) |>
    select(term, suid_count_2015_2019, e_pov_2014)
```

```
# A tibble: 1 × 3
  term           suid_count_2015_2019 e_pov_2014
  <chr>                         <dbl>      <dbl>
1 e_mobile_2014               -0.0548    -0.0298
```

This resulted in only one hit: `e_mobile_2014_2014` - Estimate of the number of mobile homes, 2010-2014 ACS. Due to low correlation with SUID too, this was unlikely to be very useful, but we added it to our additively screened variables just in case.

Here we trialed adding each of the remaining prospective variables to the model with total population and poverty, then compared performance:

```
totpop_pov_sngpnt <-
    MASS::glm.nb(
        suid_count_2015_2019 ~ e_sngpnt_2014 + e_totpop_2014 + e_pov_2014,
        data = suid
    )

totpop_pov_minrty <-
    MASS::glm.nb(
        suid_count_2015_2019 ~ e_minrty_2014 + e_totpop_2014 + e_pov_2014,
        data = suid
    )

totpop_pov_crowd <-
    MASS::glm.nb(
        suid_count_2015_2019 ~ e_crowd_2014 + e_totpop_2014 + e_pov_2014,
        data = suid
    )

totpop_pov_mobile  <-
    MASS::glm.nb(
        suid_count_2015_2019 ~ e_mobile_2014 + e_totpop_2014 + e_pov_2014,
        data = suid
    )

compare_performance(
    totpop_pov, totpop_pov_sngpnt, totpop_pov_minrty, totpop_pov_crowd, totpop_pov_mobile
    metrics = "common",
    rank = TRUE
) |>
    print_html()
```

Comparison of Model Performance Indices						
Name	Model	Nagelkerke's R2	RMSE	AIC weights	BIC weights	Performance-Score
totpop_pov_crowd	negbin	0.70	17.49	0.842	0.647	75.00%
totpop_pov	negbin	0.67	10.97	0.072	0.287	37.60%
totpop_pov_mobile	negbin	0.67	10.91	0.027	0.020	25.89%
totpop_pov_minrty	negbin	0.67	12.03	0.030	0.023	20.95%
totpop_pov_sngpnt	negbin	0.67	13.22	0.029	0.023	16.50%
NA						

The model adding crowded households performed best.

Here we trialed adding each of the remaining prospective variables to the model with total population, poverty, and crowded households, then compared performance:

```
totpop_pov_crowd_sngpnt <-
    MASS::glm.nb(
        suid_count_2015_2019 ~ e_sngpnt_2014 + e_crowd_2014 + e_totpop_2014 + e_pov_2014,
        data = suid
    )

totpop_pov_crowd_minrty <-
    MASS::glm.nb(
        suid_count_2015_2019 ~ e_minrty_2014 + e_crowd_2014 + e_totpop_2014 + e_pov_2014,
        data = suid
    )

totpop_pov_crowd_mobile <-
    MASS::glm.nb(
        suid_count_2015_2019 ~ e_mobile_2014 + e_crowd_2014 + e_totpop_2014 + e_pov_2014,
        data = suid
    )


compare_performance(
    totpop_pov_crowd, totpop_pov_crowd_sngpnt, totpop_pov_crowd_minrty, totpop_pov_crowd_
    metrics = "common",
    rank = TRUE
) |>
    print_html()
```

Comparison of Model Performance Indices						
Name	Model	Nagelkerke's R2	RMSE	AIC weights	BIC weights	Performance-Score
totpop_pov_crowd	negbin	0.70	17.49	0.324	0.713	68.69%
totpop_pov_crowd_minrty	negbin	0.71	26.35	0.437	0.185	55.08%
totpop_pov_crowd_mobile	negbin	0.70	17.50	0.119	0.051	27.49%
totpop_pov_crowd_sngpnt	negbin	0.70	18.34	0.120	0.051	22.68%
NA						

Here we reached the point where there was not enough performance boost to overcome risk of overfitting when adding any of the remaining variables.

Here we compared all step-wise iterations of the viable model to each other:

```
compare_performance(
    totpop_pov_crowd, totpop_pov, totpop,
    metrics = "common"
) |>
    print_html()
```

Comparison of Model Performance Indices					
Name	Model	AIC (weights)	BIC (weights)	Nagelkerke's R2	RMSE
totpop_pov_crowd	negbin	566.9 (0.92)	583.3 (0.69)	0.70	17.49
totpop_pov	negbin	571.8 (0.08)	585.0 (0.31)	0.67	10.97
totpop	negbin	649.3 (<.001)	659.2 (<.001)	0.18	2.85

The model with all 3 variables had the best AIC, BIC, and Nagelkerke's R^2 metrics, but suffered relative to the others on RMSE. In balance, it still seemed like the 3-variable model was the best choice.

Conclusion

To recap, our final model used a negative binomial generalized linear model, did not adjust for zero-inflation, did include an adjustment for overall population, and included the following additional predictors:

Theme 1 - Socioeconomic: `e_pov_2014` - Estimate of people living below the poverty line, 2010-2014 ACS

Theme 4 - Housing Type/Transportation: `e_crowd_2014` - Estimate of households with more occupants than rooms, 2010-2014 ACS
